# Supplementary figures and images for: Serine/threonine protein kinase phosphorylation of DosR alters target gene transcription mechanics and regulates Mycobacterium tuberculosis response to nitric oxide stress
Source: PLoS Genet. 2026 Feb 12;22(2):e1012043. doi: 10.1371/journal.pgen.1012043 (PMC12919924; doi:10.1371/journal.pgen.1012043)

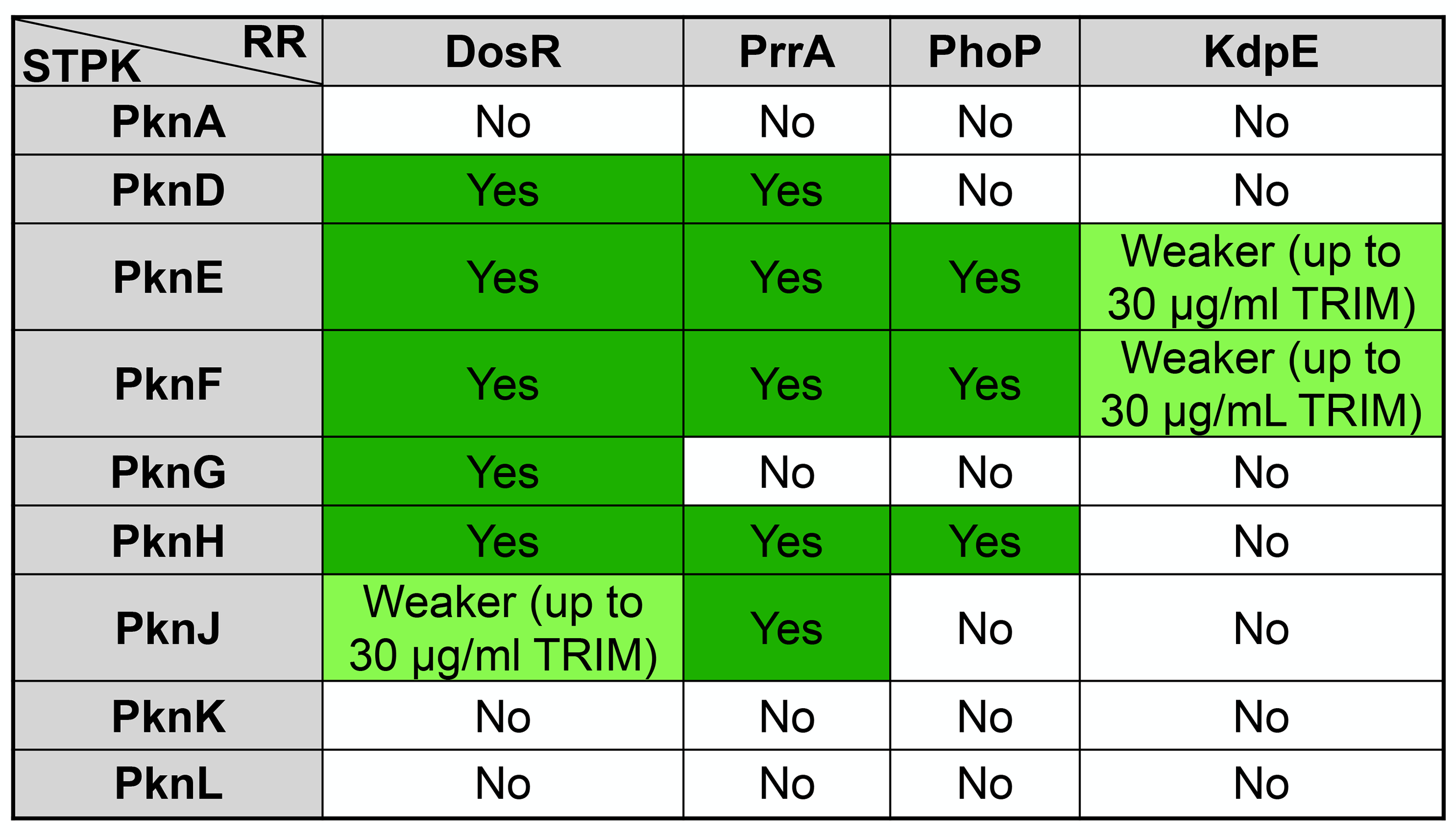

Supplement: S1 Fig — Summary table of interactions between the RRs PrrA, DosR, PhoP, and KdpE with various STPKs (kinase domains only for all except PknG and PknK) as determined by M-PFC assays. Results are representative of 2–3 independent experiments. (TIF) [file pgen.1012043.s001.tif]

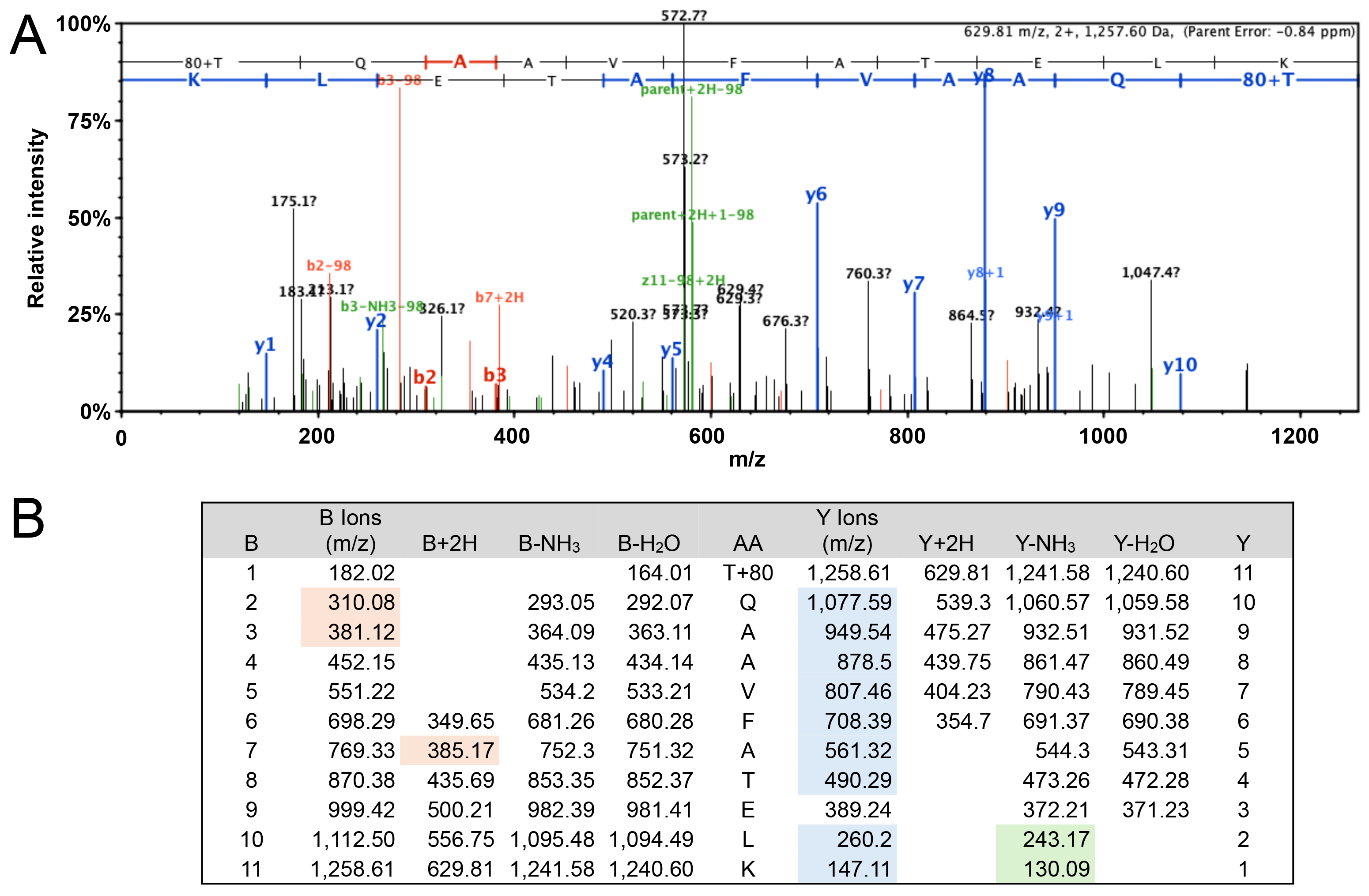

Supplement: S2 Fig — (A) MS/MS spectrum of the peptide tQAAVFATELK (m/z 629.81, z = 2). Observed b and y ions, along with neutral-loss fragments (-98 Da), are indicated. (B) Fragmentation map displaying detected ions (red = b; blue = y; green = neutral-loss/derived fragments) confirming site localization at T198. Spectra were searched using Mascot v2.8.3 and MSFragger in Scaffold 5.3.3 (precursor tolerance = 10 ppm; fragment tolerance = 0.05 Da; fixed Cys + 57.02; variable phospho +79.97 [STY]; enzyme = trypsin, ≤ 3 missed cleavages; peptide/protein FDR < 1%; peptide probability > 90%). (TIF) [file pgen.1012043.s002.tif]

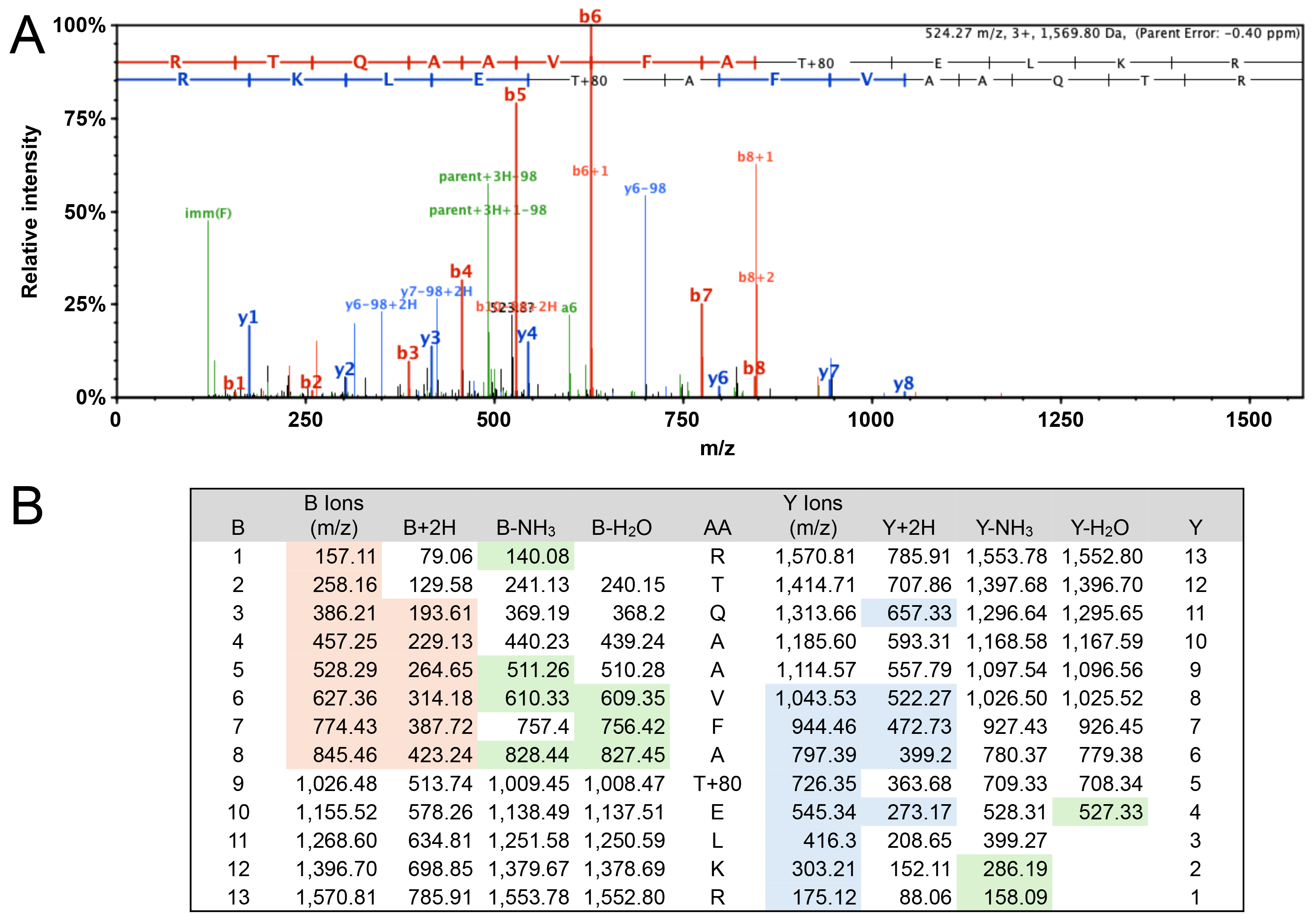

Supplement: S3 Fig — (A) MS/MS spectrum of the peptide RTQAAVFAtELKR (m/z 524.27, z = 3). Observed b and y ions, along with neutral-loss fragments (-98 Da), are indicated. (B) Fragmentation map displaying detected ions (red = b; blue = y; green = neutral-loss/derived fragments) confirming site localization at T205. (TIF) [file pgen.1012043.s003.tif]

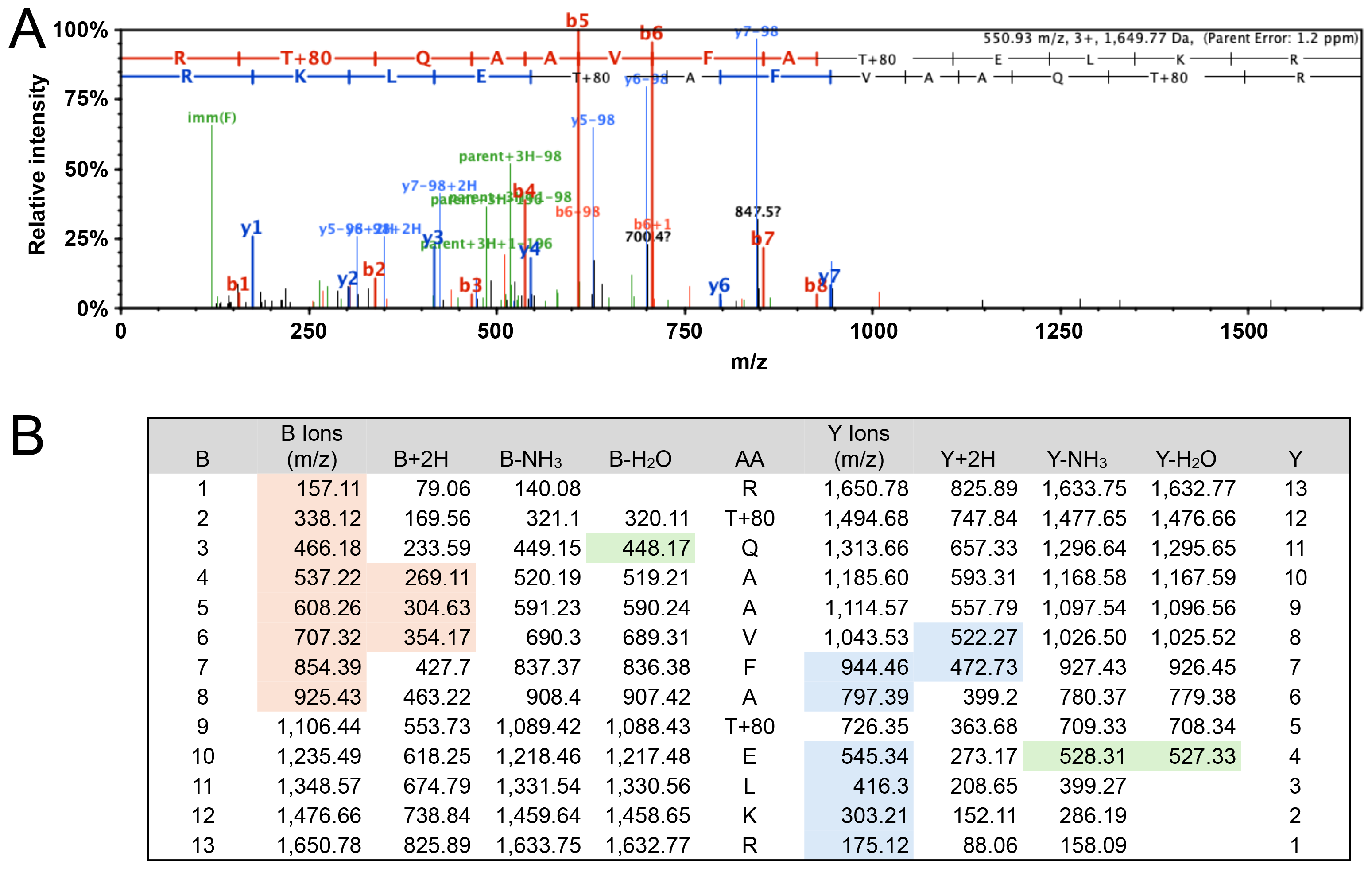

Supplement: S4 Fig — (A) MS/MS spectrum of the peptide RtQAAVFAtELKR (m/z 550.93, z = 3). Observed b and y ions, along with neutral-loss fragments (-98 Da), are indicated. (B) Fragmentation map displaying detected ions (red = b; blue = y; green = neutral-loss/derived fragments) confirming site localization at T198 and T205. (TIF) [file pgen.1012043.s004.tif]

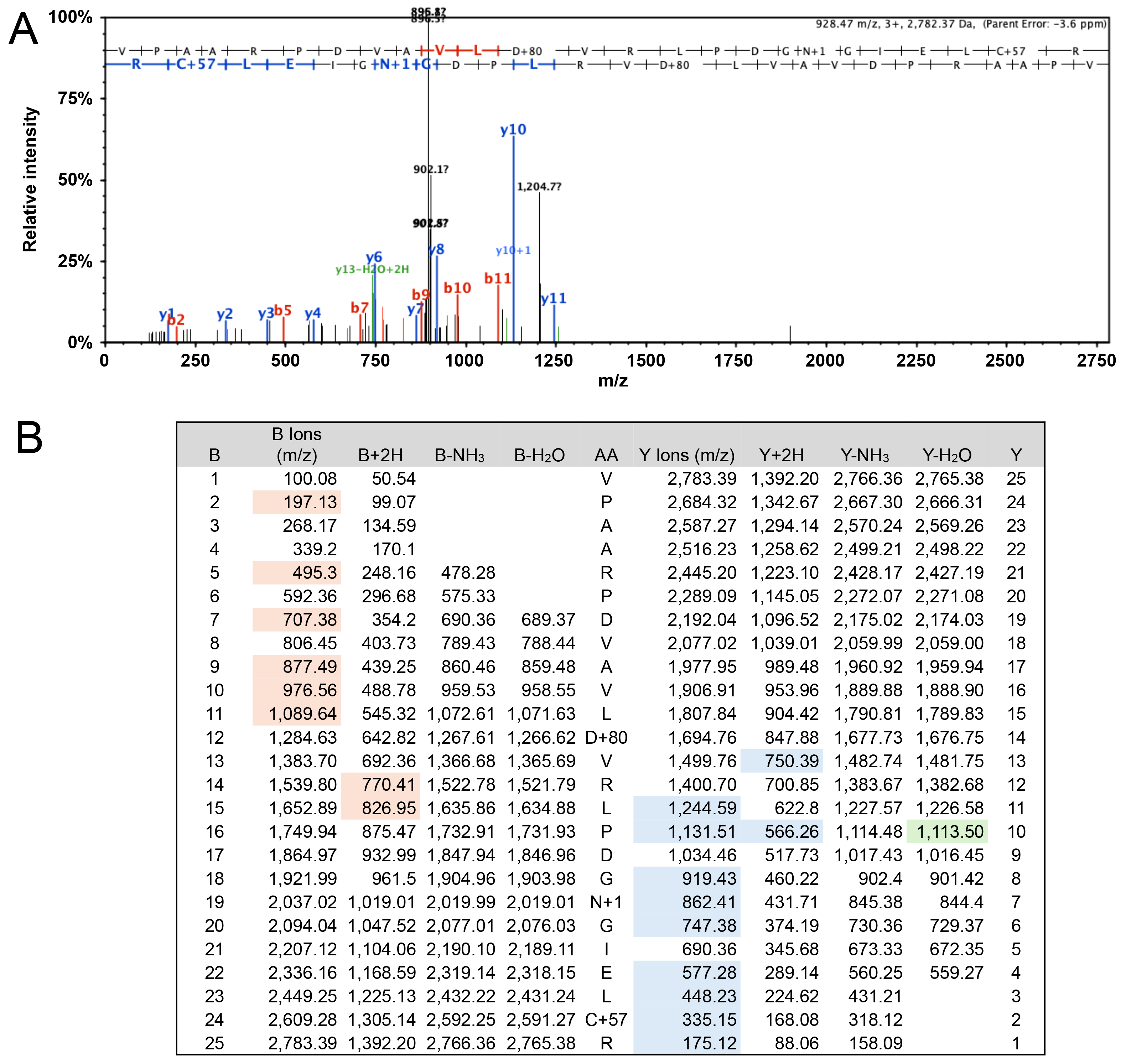

Supplement: S5 Fig — (A) MS/MS spectrum of the peptide VPAARPDVAVLdVRLPDGnGIELcR of (m/z 928.5, z = 3). Observed b and y ions, along with neutral-loss fragments (-98 Da), are indicated. (B) Fragmentation map displaying detected ions (red = b; blue = y; green = neutral-loss/derived fragments) confirming site localization at D54. (TIF) [file pgen.1012043.s005.tif]

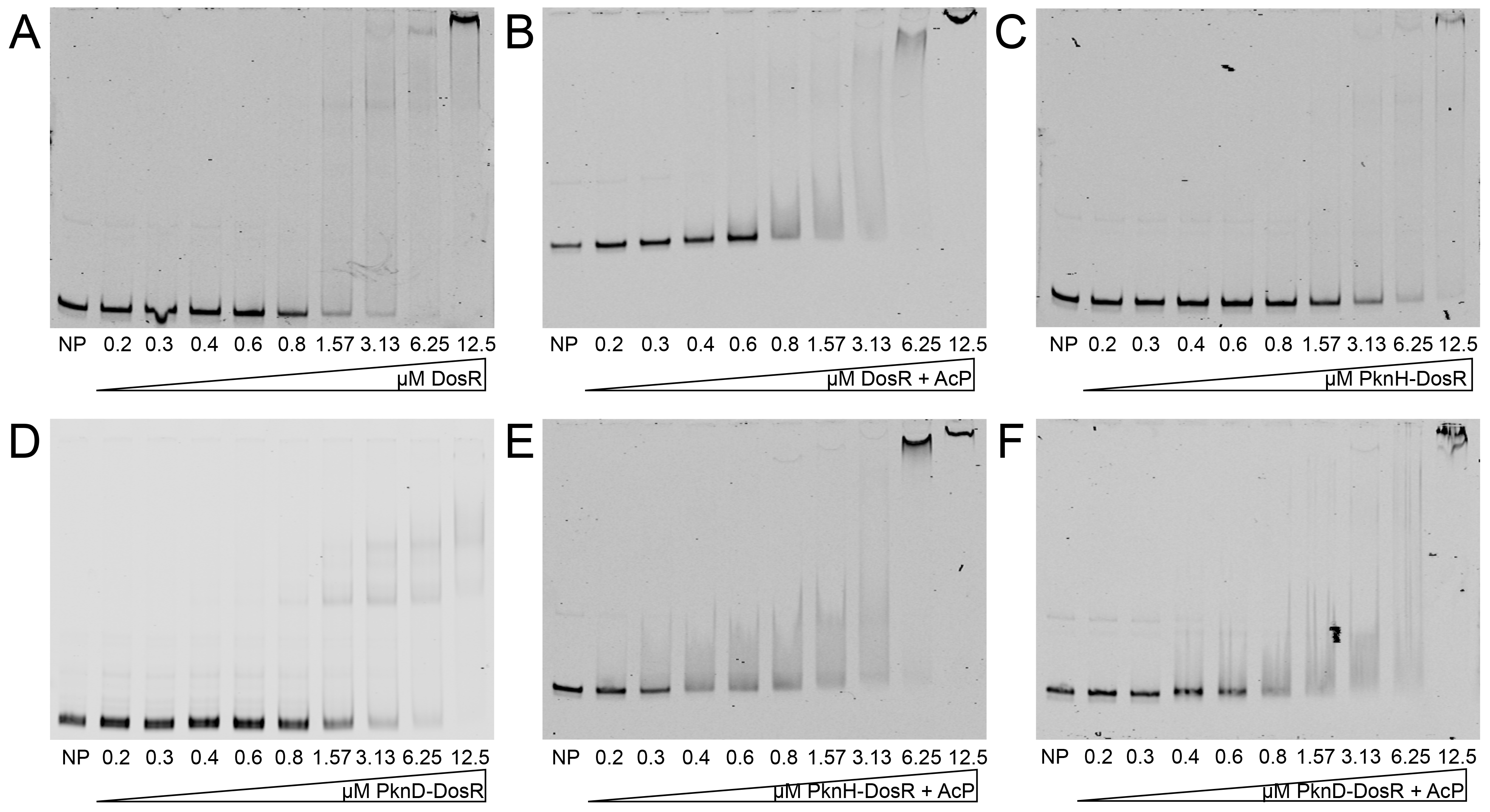

Supplement: S6 Fig — Electrophoretic mobility shift assays (EMSAs) using purified recombinant C-terminally 6x-His-tagged DosR and IRDye 700-labeled probes for the fdxA promoter are shown. A control with no protein (“NP”) added is shown for each gel. DosR was added at indicated concentrations for all other lanes. 40 fmoles of fdxA promoter DNA was used in each reaction. EMSAs shown are as follows: (A) untreated DosR, (B) DosR incubated with 50 mM acetyl phosphate (AcP), (C) DosR phosphorylated “on-bead” with 1 µM PknH, (D) DosR phosphorylated “on-bead” with 1 µM PknD, (E) DosR phosphorylated “on-bead” with 1 µM PknH, then purified and incubated with 50 mM AcP, and (F) DosR phosphorylated “on-bead” with 1 µM PknD, then purified and incubated with 50 mM AcP. Data are representative of 3 independent experiments. (TIF) [file pgen.1012043.s006.tif]

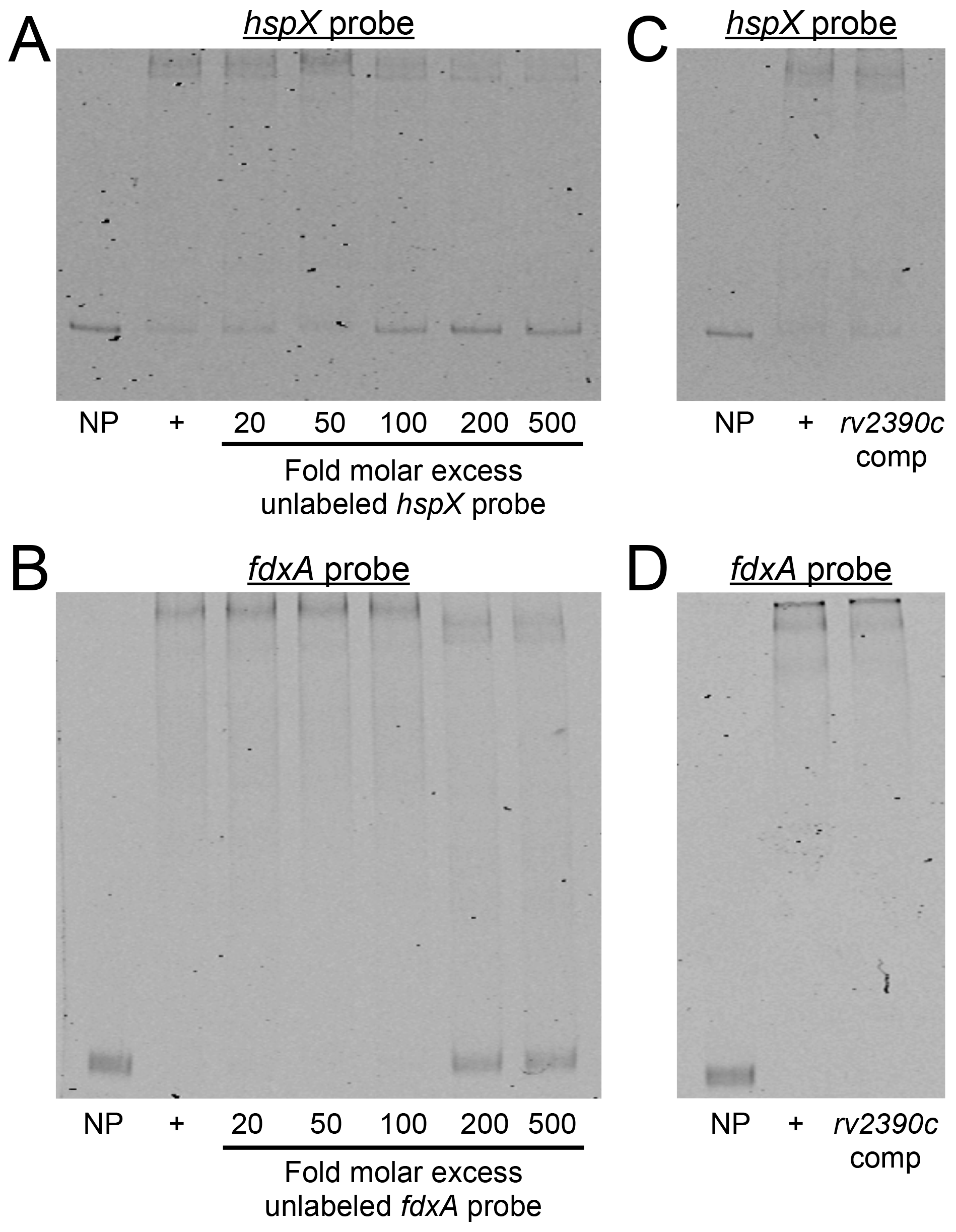

Supplement: S7 Fig — Electrophoretic mobility shift assays (EMSAs) using purified recombinant C-terminally 6x-His-tagged DosR and IRDye 700-labeled probes for the hspX promoter (A and C) and the fdxA promoter (B and D) are shown. A control with no protein (“NP”) added is shown for each gel. Purified DosR was added at 1.57 µM for all other reactions in the hspX promoter EMSAs, and at 6.25 µM for the fdxA promoter EMSAs. 1 fmole of each labeled promoter DNA was used in each reaction. “+” are reactions with DosR and the indicated labeled probe, with no competitor unlabeled probe. Where noted, unlabeled specific competitive hspX (A) or fdxA (B) probes were added at the indicated fold molar excess. In (C) and (D), unlabeled non-specific rv2390c promoter probes were added at 200-fold molar excess for reactions in the “rv2390c comp” lane. (TIF) [file pgen.1012043.s007.tif]

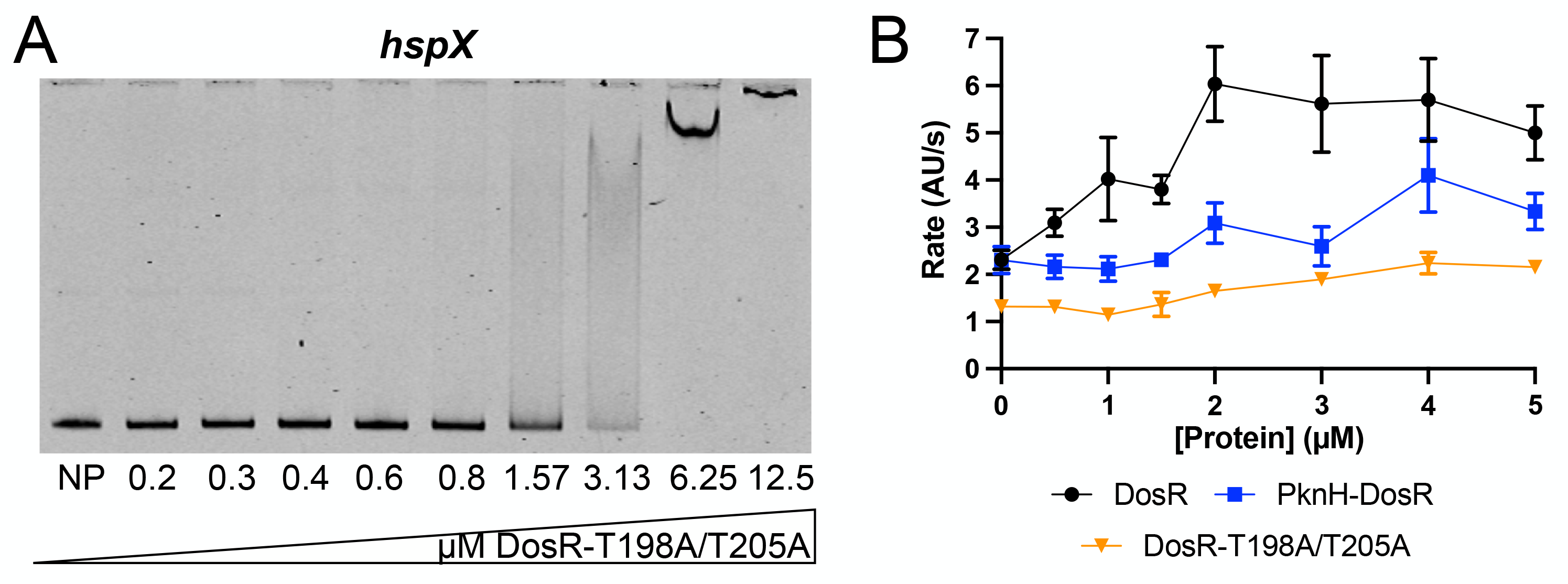

Supplement: S8 Fig — (A) shows an EMSA using purified recombinant C-terminally 6x-His-tagged DosR-T198A/T205A and IRDye 700-labeled probes for the hspX promoter. A control with no protein (“NP”) added is also shown. DosR-T198A/T205A was added at indicated concentrations for all other lanes. 40 fmoles of hspX promoter DNA was used in each reaction. Data are representative of 3 independent experiments. (B) shows a Spinach RNA aptamer assay run with the fdxA promoter with different concentrations of indicated DosR protein. The WT DosR and PknH-phosphorylated DosR (“PknH-DosR) data are as shown in Fig 4A. Fluorescence (arbitrary units, “AU”) was tracked over time on a plate reader, and steady-state rate calculated. Data are shown as means ± SEM from 2-8 experiments. The numerical data underlying the graph shown in this figure are provided in S1 Data. (TIF) [file pgen.1012043.s008.tif]
